# Local diagnostic reference levels for routine chest X-ray examinations at a public sector hospital in central South Africa

**DOI:** 10.4102/hsag.v26i0.1622

**Published:** 2021-08-17

**Authors:** Maurice Junda, Henra Muller, Hesta Friedrich-Nel

**Affiliations:** 1 Department of Clinical Sciences, Faculty of Health and Environmental Sciences, Central University of Technology, Bloemfontein, South Africa

**Keywords:** diagnostic reference levels, DRLs, chest X-ray, entrance surface air kerma, ALARA, digital radiography

## Abstract

**Background:**

Dose optimisation is a radiation protection guideline recommended by the International Commission on Radiological Protection (ICRP) for adherence to the ‘as low as reasonably achievable’ (ALARA) principle. Diagnostic reference levels (DRLs) are used to optimise patients’ radiation protection for diagnostic and interventional procedures and are particularly useful for frequently performed examinations such as chest X-rays.

**Aim:**

To establish the local diagnostic reference levels (LDRLs) for routine chest X-rays.

**Setting:**

Public sector hospital, Northern Cape province, South Africa.

**Methods:**

Sixty patients referred for chest X-rays fulfilling the inclusion criteria participated in this study. Patients were ≥ 18 years of age and weighed between 60 kg and 80 kg. Consent for participation was obtained. The entrance skin air kerma (ESAK) was measured by using the indirect method recommended by the International Atomic Energy Agency (IAEA). Statistical software (SAS version 9.2) was used to determine the LDRLs for chest X-rays in three different rooms. In two rooms, computed radiography (CR) was used and the other one was a digital radiography (DR) unit. The LDRL values at the research site were compared with various published international values.

**Results:**

LDRLs for chest X-rays were established. The CR LDRL value for the posteroanterior (PA) chest projection was higher than the DR (flat panel detector [FPD]) LDRL value. The LDRLs of the PA chest projections were 0.3 mGy for CR and 0.2 mGy for DR. The lateral (LAT) chest projection LDRL value was 0.8 mGy for both CR and DR (FPD) projections. The resultant LDRL between rooms at the research site was 0.3 mGy for PA 0.3 mGy and 0.8 mGy for LAT chest projections.

**Conclusion:**

The LDRLs for chest X-rays established at this research site were lower than internationally reported DRLs. We recommend that LDRLs for routine chest X-rays should be repeated every 3 years, according to the ICRP.

**Contribution:**

Currently, no established or published DRL values prescribed by the Directorate of Radiation Control (DRC) are available in South Africa. The LDRLs established for routine chest X-ray examinations at this research site can serve as a guideline for the establishment of DRL values for other anatomical regions at the research site and other radiology departments in the country.

## Introduction

The use of ionising radiation in medicine is currently regarded as the most prominent contributing factor to human exposure to radiation (Wambani et al. 2015). Ionising radiation has the potential to break apart the biological essential molecules such as deoxyribonucleic acid (DNA) in exposed cells and cause harm. Consequently, the amount of radiation received by patients undergoing X-ray examinations needs to be quantified to estimate the possibility of harm (Shahbazi-Gahrouei 2006). Given these circumstances, the practice of justification, optimisation of the radiation dose and diagnostic reference levels (DRLs) promote optimal radiation protection.

Dose optimisation is one of the radiation protection guidelines recommended by the International Commission on Radiological Protection (ICRP) and ensures adherence to the ‘as low as reasonably achievable’ (ALARA) principle. Furthermore, the ICRP recommends the practice of DRLs to optimise and monitor radiation dose. Determining DRLs and comparing these values with published data will ensure that the ALARA principle is achieved in radiology departments (ICRP 2007). If the locally determined DRLs are higher than the published data, these values should be investigated. A lower determined DRL is an indication that the optimal amount of radiation was used to produce radiographs of acceptable image quality.

## Literature review

Diagnostic reference levels are used to optimise the radiation protection of patients for diagnostic and interventional procedures and are particularly useful for more common X-ray examinations, which may involve high doses or are performed frequently, amongst others chest and lumbar spine X-rays (European Commission [EC] 1999). Optimisation should be prioritised according to the potential risk of stochastic effects on patients, and those resulting in substantial doses to radiosensitive organs should be addressed as a matter of priority (ICRP 2017).

The ICRP (2017) defined a DRL value as:

[*A*] selected level of a radiation dose quantity for broadly defined types of equipment for typical examinations for groups of patients within an agreed weight range or, in certain specific circumstances, a standard phantom. (p. 15)

When performing standard procedures that require acceptable diagnostic and technical practice, these levels should not be exceeded (EC 1999). In the radiology department, the DRLs of different radiological examinations are methods for optimisation of the radiation dose to which patients are exposed without compromising diagnostic image quality. Diagnostic reference levels can be used to detect abnormally high doses not making a significant contribution to the clinical findings of a medical examination (ICRP 2017).

In South Africa, the Directorate of Radiation Control (DRC) of the Department of Health defines DRLs and provides the objectives of DRLs in the code of practice for users of medical X-ray equipment (DRC 2015). The DRC has developed procedures to obtain DRLs. However, only a limited number of South African studies addressing this topic has been published to date. These include a study conducted on six different radiography examinations at the Charlotte Maxeke Hospital in Johannesburg (Nyathi et al. 2009), a study on the DRLs for the cardiology units at Universitas Academic Hospital in Bloemfontein (Makosa & Conradie 2015) and a more recent study on DRLs during intracranial aneurysm coil embolisation at a tertiary academic hospital in Cape Town (Peter 2019). Therefore, the limited information published in South Africa confirms the lack of current research and supports the necessity of this study on DRLs for routine chest X-rays in particular.

Rapid advances in imaging modalities occurred over the past decade, such as the change from film/screen technology to digital radiography (DR). One of the advantages of DR is the dynamic range, representing the range of X-ray exposure from which a meaningful image can be acquired. Digital detectors have a wider dynamic range, which eliminates the risk of a failed exposure in clinical practice. The detector function improves as radiation exposure increases without saturation seen in film/screen imaging. However, care must be taken not to overexpose the patient by applying more radiation than required for a diagnostically sufficient image (Körner et al. 2007). These technological changes have also been experienced in the specific imaging department in the Northern Cape province of South Africa, where the research was conducted and necessitated patient dose assessment for diagnostic procedures in clinical practice (Torres et al. [2004] cited by İnal & Ataç 2014).

Chest radiography is an X-ray projection of the chest used to diagnose conditions affecting the chest and surrounding structures and is anecdotally accepted to be the most commonly performed X-ray examination worldwide (Bell & Jones n.d.). This is further confirmed by the fact that general practitioners in the UK most frequently request chest radiographic imaging (NHS England 2019). The reasons why chest X-rays are performed so often include the ease of executing a chest radiograph, less radiation exposure to the patient and lower cost when compared with computed tomography (CT) scans (Raoof et al. 2012). Numerous conditions can be diagnosed by means of chest radiography, such as those involving the chest wall, bones of the thorax and structures within the thorax, including the lungs, heart and large blood vessels. Chest radiographs are also used to diagnose infectious diseases of the respiratory tract and screen for job-related lung diseases in industries such as mining, where workers are exposed to the inhalation of harmful substances (Ibrahim et al. 2014).

It is important to establish local diagnostic reference levels (LDRLs) for chest X-ray examinations because the chest contains two radio-sensitive organs, namely the thyroid and the breast tissue. Optimisation efforts will reduce the potential risk of stochastic effects on the patients (ICRP 2017). The aim of the study was to establish the LDRLs for routine chest X-ray examinations at a public sector hospital in the Northern Cape province, South Africa.

## Methods

The research was conducted in three phases, namely Phase 1: quality control (QC); Phase 2: participant selection, chest imaging procedure and a pilot study; and Phase 3: entrance skin air kerma (ESAK) calculation.

### Phase 1: Quality control

An appropriately trained professional, registered with the Health Profession Council of South Africa (HPCSA) as a medical physicist, ensured that all of the 3-monthly or yearly QC tests on all the stationary X-ray machines used for chest examinations at this hospital were performed and that the results met the requirements of the DRC (2015).

### Phase 2: Chest imaging procedure and participant selection

Radiographers registered with the HPCSA assisted in documenting the weight, patient measurement (thickness), exposure parameters, source image distance (SID) and room number on a data sheet. A pilot study was conducted to test the validity of information from the datasheet and a programmed Microsoft Excel spreadsheet was used to calculate ESAK. Four radiographers participated in the pilot study and followed the prescribed procedure to collect data, verify the data and avoid pitfalls. Data of the first 10 patients who met the inclusion criteria were captured. Their data were included in the final analysis, as no changes between the pilot study (data collection) and the main study were required.

The characteristics of the participants in this research study were based on the recommendations of the ICRP. As proposed by the ICRP (2017), standard size patients were selected for this study. Standard size patients are those whose weight falls within approximately 10 kg of the mean population being considered. According to the most recent publication by the Department of Health in South Africa (Shisana et al. 2014), the mean weight of the population is 70 kg. Thus, patients who weighed between 60 kg and 80 kg were selected to participate in this study. The patient had to be referred for a routine chest X-ray examination, be 18 years and older and agreed to sign the informed consent form. The first 60 patients who met these criteria were included in the study. According to the ICRP (2017), the minimum number of patients required to determine the DRL of any examination for a radiology department is 20 patients. In the light of this recommendation, the data of 20 patients that met the inclusion criteria for each X-ray room was captured during this study.

### Phase 3: Entrance skin air kerma calculation

The indirect calculation method is the most straightforward approach to adopt for measuring patient radiation dose as it involves less additional equipment, but it does require a measurement of the X-ray unit output. An indirect method of measuring patient radiation dose is through the evaluation of ESAK from measured kilovolt peak (kVp), milliampere-second (mAs), focus-to-skin distance (FSD) and X-ray tube output, by using an empirical formula (Essien, Okonlnyang & Egbe 2016).

The detector air kerma (*k* [*d*]) values at different kVp settings were first determined by using a calibrated detector (RaySafe X2 R/F; Fluke Biomedical, Cleveland, OH). The detector was placed at 1-m focus detector distance (FDD) on top of the X-ray machine table in the central beam axis. The *k* (*d*) was measured at different kVp settings in 10 kVp steps from 40 kVp to 125 kVp, while the mAs was kept constant at 20 mAs (Taha et al. 2015). The mAs, kVp and *k* (*d*) were recorded by a medical physicist. This process was repeated three times for the same setting after which the mean *k* (*d*) was determined. The *k* (*d*) was measured three times to eliminate a false reading from the detector. If the number was too high/low compared with the other two numbers, then the high/low value could easily be eliminated. This is an observation method to prevent false readings from affecting the quality of the data collected. The mean *k* (*d*) helps to minimise the error of each *k* (*d*) value (personal communication; medical physicist, 20 May 2021).

The X-ray tube output (*y* [*d*]) was determined by means of equation 1 (International Atomic Energy Agency [IAEA] 2007).

Y (d) = k (d)/mAs [Eqn 1]

A graph was plotted with the X-ray tube output on the y-axis and the kVp settings on the x-axis. An equation was created from this graph. This equation was used to determine the X-ray tube output at different kVp settings. The incident air kerma patient exposure was directly calculated by using the tube efficiency and the inverse square law as follows:

$$K_{i}\;=\;Y\;(d)\;\times \text{ mAs }\times \;{\left(d/[d_{\text{FTD}}\;-\;p_{t}]\right)}^{2}$$ [Eqn 2]

where K_i_ is the incident air kerma, d is the distance between the detector and the tube spot, *d*_FTD_ is the distance between the tube focal spot and the table and *p*_*t*_ is the patient thickness at irradiation site (Rasuli et al. 2016).

The entrance skin air kerma was determined by the product of the calculated values of the incident air kerma (dose free in air) and the backscatter coefficient (BSC). The BSC is the conversion that relates the incident air kerma to the ESAK. The BSC is defined as the quotient between the absorbed dose on the surface of the patient (skin) to the absorbed dose at the same point in space in the absence of the patient. This parameter provides the factor by which the radiation dose at a determined point in air is increased by radiation scattered to the same point from the patient (Rasuli et al. 2016). The BSC varies between approximately 1.3 and 1.4 for general radiography, with the exception of mammography. A single average value of 1.35 can therefore be employed in most situations without appreciable error (EC 1996).

### Ethical considerations

Approval to conduct the study was obtained from the Health Science Research Ethics Committee (HSREC; reference number: UFS-HSD2018/1610/2603) at the University of the Free State, the deputy manager of the radiology department of the government hospital in the province and the Northern Cape Provincial Department of Health. Participants provided informed consent to be included in the study. The research was conducted according to the ethical guidelines and principles of the International Declaration of Helsinki (World Health Organization [WHO] 2001).

## Results and discussion

The results are presented in table format. Table 1 illustrates the specifications of the X-ray machines used for general radiography at the research site. Rooms 1 and 2 are computed radiography (CR) units, whereas Room 3 is a DR unit. The total filtration of these machines is less than the EC requirement, which is ≥ 3 mm Al (EC 1999). According to Martin (2007), the 2.5 mm Al filtration meets the minimum requirement for an X-ray machine. The grid ratios of two of the X-ray machines were greater than the required standard of the EC, which is 10:1 (EC 1999).

**TABLE 1:** Specifications of the X-ray machines at the research site.

Specifications	Room 1	Room 2	Room 3
Make	Shimadzu	Siemens	Philips
Model	UD 150 V-40	Polydoros IT	Digital Diagnost TH
Total filtration	2.5 mm Al at 70 kV	2.5 mm Al at 70 kV	2.5 mm Al at 70 kV
Exposure setting	Manual	Manual/automatic	Manual/automatic
Grid ratio	-	17:1	12:1

kV, kilovoltage; mm, millimetre; –, no data available.

### Number of patients performed with computed radiography and digital radiography (flat panel detector) system in each room

Of the 60 patients included in the study, 20 patients’ data were collected from each X-ray room used in the study. A total of 40 patients were radiographed with the CR system in Rooms 1 and 2. The remaining 20 patients were radiographed in Room 3 by means of a DR (flat panel detector [FPD]) system. All 60 patients were radiographed for both posteroanterior (PA) and lateral (LAT) projections.

As shown in Table 2, the mean weight of the patients included in this research study was 69.6 kg, which compared well with the mean weight of 70 kg of the South African population (Shisana et al. 2014). The mean weight of the patients in this study confirms that the criterion for weight range as prescribed by the ICRP (2017) was met. The body thickness measured was used to enable the radiographers to select the correct exposure parameters from the exposure chart and to calculate the radiation dose the patient received from each projection. The mean thickness measurements of the participants were 23.5 cm and 29.2 cm for PA and LAT chest projections, respectively.

**TABLE 2:** Patients’ weight and thickness for posteroanterior and lateral chest projections (*n* = 60 patients).

Variable	Posteroanterior chest projections				Lateral chest projections			
	Mean	Median	Min	Max	Mean	Median	Min	Max
Weight (kg)	69.6	70.5	60.0	80.0	69.6	70.5	60.0	80.0
Thickness (cm)	23.5	23.0	18.0	31.0	29.2	30.0	18.0	37.0

Min, minimum; Max, maximum.

The ideal range for mAs and kVp is zero, which can only be achieved when all the radiographers in a radiology department use similar exposure factors for patients with the same weight and thickness to obtain radiographs. However, this is highly unlikely, because radiographers in a radiology department use varying exposure factors because of differences in patient sizes. As the range of patient sizes increases, so will the distribution of exposure settings.

As shown in Table 3, a wide range of kVp and mAs values were found in this study. The kVp ranges were 23 (102.0–125.0) kVp for the PA chest projection and 22 (103.0–125.0) kVp for the LAT chest projection. The mAs ranges were 4.6 (1.0–5.6) mAs and 16.4 (3.4–19.8) mAs for the PA and LAT chest projections, respectively. The wide ranges of kVp and mAs were attributed to the variation in patients’ weight and thickness. The weight range was 20 kg, and the thickness range were 19 (18.0–37.0) cm for LAT chest projections and 13 (18.0–31.0) cm for PA chest projections. As the patient’s weight and thickness increased, the exposure parameters also increased, and vice versa. The wide range of the radiographic parameters could also be attributed to the radiographer’s skill, knowledge and training, and the fact that both manual and automatic exposures were used in this study.

**TABLE 3:** Radiographic parameters for posteroanterior and lateral chest projections at the research site (*n* = 60 patients).

Variable	Posteroanterior chest projections				Lateral chest projections			
	Mean	Median	Min	Max	Mean	Median	Min	Max
mAs	3.6	4.0	1.0	5.6	9.7	9.7	3.4	19.8
kVp	113.7	112.5	102.0	125.0	117.0	117.0	103.0	125.0
SID	178.5	180.0	150.0	180.0	178.5	180.0	150.0	180.0

Min, minimum; Max, maximum; mAs, milliampere-second; kVp, kilovoltage peak; SID, source image distance.

A total of 120 images were included in this study. Out of the 120 images, six images were taken at an SID of 150 cm. The other images were taken at an SID of 180 cm. These SIDs were within the range of 180 (140–200) cm recommended by the EC for chest X-ray examinations (EC 1996).

### Image quality

The exposure index (EI) and radiographic criteria for chest X-rays were applied to ensure that the radiographs used to calculate ESAK and DRL were of adequate image quality. The EI value of each chest image was within the range prescribed by the manufacturer of the X-ray machine for the anatomical area. The EI range for Room 1 and Room 2 was 172–344 and 300–500 for Room 3.

All the chest radiographs of the patients included in the study were of an acceptable standard (adequate visualisation of radiographic anatomy through correct positioning) and had acceptable image quality for diagnosis that met the image criteria required by the EC guidelines. These criteria were the prerequisites for the image standard for patient data to be included in this research study. As illustrated in Table 4, the mean ESAK values of 0.2 mGy for CR and 0.1 mGy for DR (FPD) were recorded for PA chest projections. The Mann–Whitney *U*-test (*p*-value 0.0001) demonstrated any significant difference between the median values of the two systems for the PA projection.

**TABLE 4:** Entrance skin air kerma (mGy) values for posteroanterior and lateral chest projections of computed radiography and digital radiography (flat panel detector) systems.

Type of radiography	Posteroanterior chest projections				Lateral chest projections			
	Mean	Median	Min	Max	Mean	Median	Min	Max
CR (*n* = 40)	0.2	0.2	0.2	0.4	0.6	0.6	0.4	0.9
DR (FPD) (*n* = 20)	0.1	0.1	0.1	0.3	0.6	0.5	0.2	1.4

Min, minimum; Max, maximum; CR, computed radiography; DR (FPD), digital radiography (flat panel detector).

The mean ESAK value of the PA chest projections of the DR (FPD) system was half the value of the CR system, which was because of dose efficiency. The dose efficiency of the DR (FPD) system was deemed two to three times more efficient than the CR system at converting dose to signal. This increased dose usage indicated that the DR (FPD) system could produce the same image quality as the CR system at a lower dose (Colbeth 2017).

The mean ESAK value recorded for both DR (FPD) and CR of the LAT chest projections was 0.6 mGy, as shown in Table 4. There was no difference in the ESAK values to demonstrate dose efficiency, as illustrated by the PA chest projection. The Mann–Whitney *U*-test (*p*-value 0.2201) confirmed that no significant difference between the median values of the CR system and DR (FPD) system was observed for the LAT chest projection.

Table 5 shows the summary that the mean ESAK values found in this study were lower than international findings reported in the literature, with the exception of a study conducted in the United Kingdom in 2010 (Hart, Hilliers & Shrimpton 2012). The calculation of the ESAK is fundamental for assessment of the dose to patients, which would help in making decisions on how the optimisation of the radiation dose can be achieved in the radiology department.

**TABLE 5:** Comparison of the entrance skin air kerma (mGy) values of this study to other international entrance skin air kerma values

Study and country	Projection	
	Chest PA	Chest LAT
**Nyathi et al. (2009); South Africa**		
Mean	0.10; 0.09†	0.23; 0.19†
Minimum	0.05; 0.05†	0.12; 0.07†
Maximum	0.20; 0.15†	0.44; 0.26†
**Hart et al. (2012); United Kingdom**		
Mean	0.12	0.48
Minimum	0.02	0.22
Maximum	1.1	1.26
**Baş Mor et al. (2018); Turkey**		
Mean	0.33	0.73
Minimum	-	-
Maximum	-	-
**Mohsenzadeh et al. (2018); Iran**		
Mean	0.6	0.85
Minimum	0.13	0.25
Maximum	1.12	1.98
**Metaxas et al. (2019); Greece**		
Mean	0.12	0.66
Minimum	-	-
Maximum	-	-
**Ahmed et al. (2020); Sudan**		
Mean	0.49	-
Minimum	0.07	-
Maximum	2.35	-
**Hoseini Motlagh et al. (2020); Iran**		
Mean	1.0‡	1.7‡
Minimum	0.2‡	0.3‡
Maximum	3.3‡	4.3‡
**Suliman (2020); Oman**		
Mean	0.14	0.49
Minimum	-	-
Maximum	-	-
**This study (2020); South Africa**		
Mean	0.2	0.6
Minimum	0.1	0.2
Maximum	0.4	1.4

PA, posteroanterior; LAT, lateral.

† , In this study, separate values were provided for two examination rooms.

‡ , In this study, entrance skin dose (ESD) values were reported, not entrance skin air kerma (ESAK).

The ESAK ranges were wide for both chest X-ray projections, with 0.3 (0.1–0.4) mGy and 1.2 (0.2–1.4) mGy for PA and LAT, respectively. These wide ESAK ranges could be linked to the wide ranges of the mAs, kVp, weight range, patients’ thickness and radiographer skill, knowledge and training (refer to Tables 3 and 4).

### Local diagnostic reference levels for posteroanterior and lateral chest projections per X-ray room

The LDRLs were the same as the third quartile, 75th percentile and upper quartile of the mean dose distribution. The LDRLs of the LAT chest projection were 0.8 mGy for Room 1 (CR unit), 0.7 mGy for Room 2 (CR unit), and 0.8 mGy for Room 3 (DR unit). For the PA chest projection, Room 3 had the lowest LDRL (0.2 mGy), while for both Rooms 1 and 2, the LDRL value was 0.3 mGy.

### Local diagnostic reference levels for posteroanterior and lateral chest projections for computed radiography and digital radiography (flat panel detector) systems

The CR LDRL value for the PA chest projection was higher than the DR (FPD) LDRL value. The LDRLs of the PA chest projections were 0.3 mGy for CR and 0.2 mGy for DR (FPD). The LAT chest projection LDRL value was 0.8 mGy for both CR and DR (FPD) projections.

The purpose of the LDRL is to optimise radiation dose to patients by identifying the superfluous dose not contributing to the clinical purpose of the imaging. This is achieved by comparing the LDRL values to international values. The LDRL values found in this study were less than the international DRLs, which implies that this research site has achieved the purpose of the LDRL for chest X-ray examinations. If the LDRL values had been higher than the international value, an investigation should have been undertaken to identify the reasons for the higher LDRLs.

As shown in Table 6, the LDRLs for chest X-ray examinations were compared with numerous DRLs published in the literature, including the international organisations IAEA and EC. The LDRL of this study (0.3 mGy) was similar to IAEA (2004) and EC (2018) for the PA chest projection. The LDRL for the LAT chest X-ray projection for this study was 0.8 mGy, notably less than the DRLs (1.5 mGy) recommended by the international organisations (EC 2018; IAEA 2004).

**TABLE 6:** Comparison of local diagnostic reference levels (mGy) of this study to internationally recommended and previously published diagnostic reference levels.

Study and country	Projection	
	Chest PA	Chest LAT
IAEA (2004)	0.30	1.50
Nyathi et al. (2009); South Africa	0.10	0.20
Hart et al. (2012); United Kingdom	0.15	0.54
Baş Mor et al. (2018); Turkey	0.35	0.78
European Commission (2018)†	0.30	1.50
Mohsenzadeh et al. (2018); Iran	0.63	1.11
Ahmed et al. (2020); Sudan	0.60	–
Hoseini Motlagh et al. (2020); Iran	1.40	2.10
Suliman (2020); Oman	0.20	0.60
This study (2020); South Africa	0.30	0.80

PA, posteroanterior; LAT, lateral; IAEA, International Atomic Energy Agency.

† , Most commonly used value.

Both the UK 2010 review and Iranian 2018 DRLs were obtained at a national level. Table 6 indicates that the LDRLs of this study were lower than those of the Iranian 2018 study (Mohsenzadeh, Deevband & Pouriran 2018), but higher than the UK 2010 review (Hart et al. 2012) for both projections.

The DRL results of the South African study conducted in 2009 were obtained from a single radiology practice in one hospital (Nyathi et al. 2009), whereas the results of the Turkish study (Baş Mor, Altinsoy & Söyler 2018) were representative of three hospitals. The LDRLs of this study for the LAT chest X-ray projections were similar to those of the Turkish study, whilst at 0.3 mGy the LDRLs of the PA chest projections in this study were lower than those found in the Turkish study (0.35 mGy) (Baş Mor et al. 2018). As shown in Table 6, the LDRLs reported here were lower than previously found in another South African study (Nyathi et al. 2009).

Since the implementation of DRLs by the ICRP in the 1980s, the methodology to calculate DRLs has changed numerous times. Before comparing a study’s DRL values to other results, the weight range used to select patients should be investigated. The method that was used to measure radiation dose to the patient and the type of radiographic system used to acquire these images should also be comparable. Therefore, it would be justified to compare the LDRLs of this study with the Iranian research findings as a similar weight range to select patients and a similar radiographic system and method to measure radiation dose to patients were used (Mohsenzadeh et al. 2018). However, when comparing ESAK values, the Iranian ESAK values were higher than our findings.

Table 6 shows a summary of this study’s LDRL values that they were lower than those of international organisations (EC 2018; IAEA 2004) and other previously published studies (Ahmed et al. 2020; Baş Mor et al. 2018; Hart et al. 2012; Hoseini Motlagh et al. 2020; Metaxas et al. 2019; Mohsenzadeh et al. 2018; Nyathi et al. 2009; Suliman 2020). The reason for this is that the radiographers at the research site used the high kVp and low mAs technique to produce chest X-ray radiographs. This technique also contributes to the reduction of the radiation dose to the patients. The patients who were selected to participate in this research weighed from 60 kg to 80 kg. The weight ranges of the comparable DRL studies were from 50 kg to 105 kg (Ahmed et al. 2020; Baş Mor et al. 2018; Hart et al. 2012; Hoseini Motlagh et al. 2020; Metaxas et al. 2019; Mohsenzadeh et al. 2018; Nyathi et al. 2009; Suliman 2020). As the body weight increases, so does the radiation dose to the patient, and vice versa. The DRL results demonstrated in Table 6 were obtained by means of mixed detector systems that included CR, DR (FPD) and film-screen system, except for the Iranian study reported previously (Mohsenzadeh et al. 2018). This study and the Iranian 2018 study made use of CR and DR (FPD) radiographic systems. The current study’s research site has a full-time-employed, HPCSA-registered medical physicist who performs QC and quality assurance (QA) as stipulated by the DRC (2015) and ensures that the X-ray machines are maintained and functioning appropriately.

## Limitations

The LDRLs for chest X-ray examinations of the patients weighing less than 60 kg or more than 80 kg were not included in this study. Not all the radiographers at the research site participated in the research study. The pathological condition of the patients was not taken into consideration when determining the DRLs.

### Recommendations

The LDRLs for routine chest X-ray examinations should be repeated after 3 years according to the ICRP recommendations (ICRP 2007, 2017).

## Conclusion

This study determined the ESAK of chest X-ray examinations for the participating patients. Next, the LDRLs of chest X-ray examinations were calculated at the research site. These LDRLs of the chest X-ray examinations were compared with numerous international organisations’ DRLs of other relevant studies. The LDRLs of chest X-ray examinations were lower than those of international organisations and some previously reported DRL values.

The LDRL values established in this specific study for PA and LAT chest radiographs compared well with the international values. This finding is an indication that the radiographers at the research site applied the ALARA principle to obtain chest radiographs. However, compliance may not necessarily indicate that all the chest radiography procedures were optimally performed with the least amount of radiation. Radiographers continuously need to look at methods to optimise radiation dose critically.

It should further be kept in mind that DRLs may vary substantially between different devices. Vanaudenhove et al. (2020) recently reported variabilities ranging between 30% and 200% among 590 radiographic X-ray devices installed in 345 medical centres in Belgium. According to the European law, these devices have to undergo QC checks annually and complete survey reports on X-ray dose are required every 3 years. Anonymised dose indicator values delivered to a minimum of 50 consecutive patients undergoing standard examinations should be provided. When a procedure is conducted on less than 50 patients, dose indicator values for all examinations performed within a 3-month period should be recorded (Vanaudenhove et al. 2020).
